# Examination of Candidates for Admission to the Dental Department of the University of Michigan

**Published:** 1879-11

**Authors:** 


### EXAMINATION OF CANDIDATES FOR ADMISSION TO THE DENTAL DEPARTMENT OF THE UNIVERSITY OF MICHIGAN.

Inquiry, concerning the examination of students, preliminary to entering the DENTAL DEPARTMENT of the UNIVERSITY OF MICHIGAN, has been so frequently made, that it is deemed proper to present here the written questions of that examination. These are required to be answered in writing in such a manner as to show a good acquaintance with the subjects embraced. In many instances there is, in addition to this, an oral examination:

1. What is language?
2. What is a word?
3. How are words classified?
4. What are the leading words in language.
5. To what do they pertain? What relation do they sustain to each other?
6. What is a noun?
7. What is a verb?
8. What is the significance of each?
9. Give the definition of the following words and the part of speech to which each belongs:

10.

*a*. Benefaction.
*b*. Benevolence.
*c*. Beneficent.
*d*. Beneficently.
*e*. Adjudicate.
*f*. Abbreviate.
*g*. Sterile.
*h*. Profoundly.
*i*. Opportunity.
*j*. Step by Step.
*k*. Hollo!
*l*. Begone.

11. Correct the following paragraph:

LECTRIC LEIGHTS AT SEE.

It is well knon from repeeted experaments with the lectric leight on, the see-shoar that it can bee sean for a remarkable distence; and easily penetrats the thickest fogg, why it has not bin adopted on ocien steemers, is a mystry for it could be werked by the Engins at a smal expence. And would sertenly mak marene colisions almost imposibal.

---

12. What is Geography?

13. What are the natural features and divisions of the earth's surface?

14. What are the artificial divisions?

15. How, by whom and for what purposes are artificial divisions made?

16. What, and where is the Equator?

17. What is latitude?

18. What is longitude?

19. What is the latitude and longitude of your home?

20. What is the horizon?

21. What is the ecliptic?

22. What are the principal countries of Europe?

23. Give their capitals.

24. Where is Cape Horn?

25. Give the name, location and elevation of the highest mountain on the earth.

26. Give the name of the highest mountain in North America.

27. Give the name, location and length of each of the three largest rivers in the world?

28. What nation possesses and occupies the largest territory in the world?

---

29. What are the three principal forms of government among the nations of the world?

30. Give an example of each.

31. Who was Napoleon Bonaparte the First?

32. For what was he noted?

33. When and where did he live?

34. When and where did he die?

35. What important wars have occurred in Europe and Asia within the last ten years?

36. In what year was American Independence declared?

37. What was the cause of the Revoluntionary War?

38. What are the three chief divisions or departments of the government of the United States?

39. What are the principal officers of each?

40. Where was the first English colony successfully planted in North America?

41. How many States and how many Territories in the United States?

42. How many inhabitants must a Territory contain before it can become a State?

---

43. What is Arithmetic?

44. What are the four primary rules of Arithmetic?

45. What is the length of the Equator, in yards, giving sixty-nine and a half miles to the degree?

46. What is the difference between the simple and compound interest of $5220 at 6 per cent. interest, per annum, for five year?

47. A cistern containing five hundred gallons of water, has a faucet which discharges forty-three gallons in fifty-five minutes, and a pipe supplying twenty-five gallons in forty minutes. How many hours, minutes and seconds will be required to empty the vessel?

48. Give the Square Root of 14400.

49. Find the Cube Root of 689213742.

50. What is a point?

51. What is a line?

52. What is an angle?

53. What is a square?

54. What is a cube?

55. What is a circle?

56. What is an acute angle?

57. What is a right angle?

58. How many right angles are contained in a square?

59. How many are contained in a circle?

60. What is a triangle?

